# ‘Why Would You Want to Do That?’ A Tribute to the ‘Exquisite Obsession’ and Legacy of Professor Dame June Clark, Health Visitor, 1931–2025

**DOI:** 10.1111/jan.70330

**Published:** 2025-10-23

**Authors:** Daniel Kelly, Sarah Cowley, Michelle Moseley, Debra Jackson

**Affiliations:** ^1^ Emeritus Professor in Healthcare Sciences Cardiff University Cardiff UK; ^2^ Emeritus Professor of Community Practice Development King's College London London UK; ^3^ Director of Programmes (Learning and Development) Institute of Health Visiting London UK; ^4^ Director of Academic Career Development, Susan Wakil School of Nursing The University of Sydney Sydney Australia

## 1 Introduction

The death of Professor Dame June Clark on May 14, 2025, led us to explore her unique legacy as a health visitor and nurse leader within the pages of this anniversary edition of the *Journal of Advanced Nursing*. This seemed apt as the results of one of her research studies appeared in the first ever edition of this Journal in 1976 with her paper ‘The role of the health visitor: a study conducted in Berkshire, England.’ Almost 50 years later, her name appears again in tribute and in thanks for all that she achieved. June was one of the post‐Second World War generation of women who took up a nursing career and changed thinking. Her impact was often ahead of its time and is still being felt today.

## 2 Early Influences

June Clark was born in Sheffield. Her father was a trades union official in the steel industry during the second World War, which may have influenced her own passion for worker's rights. Her parents were both Welsh, and she always considered Wales her home. At her recent memorial service on a very sunny day in Swansea, I (DK) met her surviving sister, who told me of their father's initial reaction when June announced her ambition to become a Registered Nurse. His response was ‘Why would you want to do that?’ June had excelled at school, and a university education had to come first. Her father said that after she had obtained a degree, she could do as she pleased.

So, June entered University College London to read Classics and graduated in 1962. Her studies of Latin and Greek took place on the UCL campus on the north side of Gower Street, London, WC1. After graduating, she crossed to the south side of Gower Street to commence nurse training at University College London School of Nursing—an attractive cruciform building of red brick that has since been refurbished and now hosts the UCL School of Medicine. Other nurse leaders and several Fellows of the Royal College also trained there, and an active UCL nursing association continues to this day. By 1963, June had become the first delegate of the Student Nurses' Association at the national Union of Students Conference. This was an early sign of her emerging leadership qualities.

June's early interest in nursing (described subsequently in her autobiography as an ‘exquisite obsession’) stayed with her for life (Clark 2016). Throughout her long and impactful career, she argued for a higher professional status to be afforded to nursing and for its potential (and subsequently also for that of Health Visiting) to be better recognised. In 1982 she was awarded a Fellowship of the Royal College of Nursing.

June was never afraid to challenge politicians, including John Major, the British Prime Minister, about the need for university education for nurses. In 1990, she was elected President of the Royal College of Nursing and became a vocal campaigner for better pay and career structure. June could be a formidable adversary and was unafraid to question decisions made by others in positions of power with whom she disagreed. Courage was never in short supply for June Clark, and sometimes arbitration was required to settle disagreements when she spoke truth to power.

Eventually, June found that it was Health Visiting that provided the vehicle for her academic talent to question and contribute to the scant evidence base; hence, her 1976 research paper was an important contribution to the newly launched *Journal of Advanced Nursing*. She completed an MPhil and then a PhD in 1985 titled ‘The process of health visiting’ and in 1990 was also appointed the inaugural Professor of Nursing at Middlesex University in London. Here again, she had to battle for resources and recognition, but her portrait hung in the university corridors long after she had moved on. She impacted many people's careers, including former ICU Sister at University College, Jan Williams, who eventually entered Middlesex as a nurse teacher and later was appointed Dean of the same School that June had helped to establish. Jan attended June's memorial service in Swansea.

The arc of June Clark's life shows how one woman achieved so much in many different areas. June can be considered a disruptor who, even in later years when living with a diagnosis of dementia, always had something to say and found a way to say it. Her contribution to meetings of Fellows of the Royal College of Nursing continued even up to the final months of her life.

However, it is for her contribution to the Health Visiting profession that she is best remembered. She saw its potential to help ensure a more equitable future for children and families. To understand why June made such an important contribution, it is important to understand the history of Health Visiting and why it had captured her attention so powerfully.

## 3 A New Breed

June Clark was among the earliest of the ‘new breed of health visitors,’ qualifying just 2 years after a completely revamped and extended 51‐week curriculum, established by the Council for the Education and Training of Health Visitors (CETHV), that began in 1965. The CETHV itself was set up in 1962, following the Jameson Report (1956), which had been tasked with clarifying the need for health visitors and their role.

This was a century after the start of British health visiting, which is usually traced to the Salford Ladies Sanitary Reform Association (later, Ladies Health Society), who agreed in 1862 to appoint ‘ordinary working women’ to visit all the homes in their allocated area, providing comprehensive help and advice on health and social problems (Dingwall 1977). The first paid health visitors were appointed in 1867; Dingwall describes how the practice spread across the country as more local Health Societies developed in response to the profound level of poverty and need, especially in large, industrialised cities. From the start, their visits were offered universally, without cost, to everyone in the local area to avoid any risk of stigma. Acceptability was fostered through a friendly, practical, and helpful approach, rather than any compulsion upon the families. There was a clear focus on the wellbeing of mothers, babies, and children from the start, but perhaps foreshadowing the ‘family visitor’ described by Clark (1976), these early workers visited the whole family, whilst developing an awareness of, and influence on, health needs across the neighbourhood to which they were allocated.

By the start of the twentieth century, health visiting was widespread in the United Kingdom and gradually shifted from a philanthropic stance to an officially endorsed and funded role, first by local, then national government. Once the role was formalised, training and qualifications became standardised, with the first register being held by the Royal Sanitary Institute (now Royal Society of Public Health), until that duty passed to the CETHV in 1962 (see Table 1, for a summary of how the training developed and has changed over the years).

**TABLE 1:** Development of health visiting: preparation and regulation.

**1862:** Manchester and Salford Ladies Sanitary Reform Association agreed to employ working women to visit homes to offer practical help, advice and education about health; this is usually cited as the start of health visiting
**Late 19th/early 20th century:** Courses of lectures run by Medical Officers of Health and various institutions throughout the country. Also, qualified women sanitary inspectors (forerunners of today's environmental health officers) were employed to undertake health visiting duties in addition to their other work.
**1890s onwards:** increasing number of certificated courses for health visitors; these were usually for 2 years, or 6 months for graduates, qualified teachers or nurses.
**1907/1915:** Birth Notification Acts laid the ground for a national service based on home visiting to newborn infants. Once local authorities started raising revenue via rates to pay for health visiting, qualifications began to be stipulated.
**1909:** Health visitors' (London) Order for London CC Area. First statutory qualification, in London area only.
**1916:** Royal Sanitary Institute (RSI; later Royal Society of Health, now Royal Society for Public Health) began co‐ordinating qualifying courses for health visitors; still 2 years or 6 months for graduates/nurses.
**1925:** Ministry of Health took over responsibility for the training of health visitors. Qualifications were definitely required for the work; a midwifery qualification was a pre‐requisite. RSI was the designated examining body.
**1929:** Local Government Act Statutory Rules and Orders (1930 No 69) laid down qualifications for health visitors and tuberculosis workers; later adjustments in Public Health Act 1936; Education Act and School Health Service Regulations 1959.
**1945:** National Standing Conference of Health Visitor Training Centres (now UK Standing Conference of Specialist Community Public Health Nursing) established.
**1948:** National Health Service (Qualifications of Health Visitors and tuberculosis visitors) Statutory Instrument Number 1415; possession of health visitor certificate confirmed as a statutory requirement for practice as a health visitor in UK. Health visitors employed by Local Authorities (Public Health) under new NHS structure.
**1950:** Royal Society of Health (now Royal Society for Public Health) revised syllabus and extended training from 6 to 9 months minimum for qualified nurses and midwives.
**1956:** Jameson Committee: recommended health visitors concentrate on health of whole family, not just babies, including health education and local communities. Led to Council for the Education and Training of Health Visitors.
**1962:** Council for the Education and Training of Health Visitors (CETHV) established as the regulating authority. They developed a curriculum for a ‘new breed of health visitor’, based on a 51‐week course (implemented 1965). Confirmed a nursing qualification as a statutory pre‐requisite for entry into health visitor training.
**1964:** National Health Service (Qualifications of Health Visitors) Regulations (para. 2a). Wording updated and statutory status of qualification confirmed in (Statutory Instrument 1972, No. 1822) and in 1973 Act, which led to local government Public Health Authorities merging into with NHS in 1974.
**1972:** Health visiting was included in the remit of the Commission on Nursing (Briggs Committee), which led to the formation of the United Kingdom Central Council for Nursing, Midwifery and Health Visiting (UKCC). Briggs recommended replacing the health visitor training with a 6‐month, non‐statutory certificate in preventive nursing, and that health visitors become known as ‘family health sisters’.
**1979:** Nurses, Midwives and Health Visitors Act 1979 established the UKCC. It became fully operational in 1983, at which time the CETHV ceased to function. The 51‐week syllabus introduced in 1965 continued until 1994.
**1983:** Health visiting register/regulation transferred from CETHV to the UKCC + health visitor education and training transferred to four National Boards (England, Northern Ireland, Scotland, Wales).
**1986:** Project 2000 proposals commit future nurse education to a ‘health’ philosophy and increased community experience at a pre‐registration level; four distinct branches of nursing with 18‐month common foundation programme. Nurse education began moving into universities, qualification at Higher Education Diploma level in first instance.
**1994:** New framework for preparing specialist practitioners as ‘Community Health Care Nurses’ to include health visiting as one of eight areas of community nursing practice; programme length reduced to a minimum of 32 weeks at degree level, at least one third of programme to be specific to health visiting.
**1998:** JM Consulting Ltd. Review of UKKC recommended that health visiting should cease to be registered as a separate profession, being regarded instead as a specialist branch of nursing.
**1999:** Fitness for practice report on pre‐registration nurse education reduced length (to 12 months) of common foundation programme; reduced health and community input in favour of more clinical nursing skills
**2001:** Nursing and Midwifery Order (Statutory Instrument 2002 No 253): laid the ground for establishing the new Nursing and Midwifery Council (NMC), closing the former (UKCC) health visiting register. As a result, the terms ‘health visitor’ and ‘health visiting’ were removed from all extant legislation.
**2004:** NMC set up with a two‐part register, to regulate nurses and midwives. A third part, attached to the nursing one, covered ‘specialist community public health nurses’ (SCPHN), to include health visitors. Training extended to 45 programmed weeks, with up to half to be shared with school and occupational health nurses.
**2022**: NMC: updated Standards of Proficiency for SCPHNs, with health visitors named as one field of practice.

## 4 The Contribution of Research

Clark's (1976) seminal work was based on her MPhil (Master's by research) study, at a time when health visiting was developing rapidly under the auspices of the CETHV. The health visiting workforce expanded throughout the 1970s, beginning to stall as the 1980s drew to a close. By that time, Clark was a well‐known pioneer in nursing and health visiting research circles, having completed her doctorate on the process of health visiting in 1985. That study drew on her interest in nursing models and theories, which were popular at the time, particularly in the US. Her application of Betty Neuman's systems model provided a description of health visiting as a continuing process, accounting for their work with whole families as ‘patients’ who were constantly interacting with their environment, coping with stresses and maintaining stability (Clark 1982, 1986).

Clark (2020) traced the loss of health visiting's influence along with their broader family role through the 1990s, the closure of the dedicated register in 2004, and subsequent sharp reduction in the workforce, to policies formed as far back as the 1970s (e.g., the Briggs Report, Department of Health and Social Security 1972). However, she recognised and was driven by the responsibility of the profession to describe and measure health visiting activities more clearly, as the main way to influence health policies. As her international profile developed through the 1990s and 2000s, Clark contributed substantially to the huge body of work on developing an international classification of nursing practice (Clark and Lang 1992, 1997; Hughes et al. 2008). She never strayed far from health visiting, though, using this work to suggest a new method for documenting health visiting practice (Clark and Mooney 2001).

Sadly, Clark's ideas were often ahead of her time, and a substantial review of health visiting at the turn of the century (Clark et al. 2000) was quietly shelved by the Welsh Government that had commissioned it. Even so, the enduring ideas of universality, acceptability, a focus on the whole family (not just mothers and babies) within a community, the stresses they face as well as supporting them to enhance health, should not be lost in today's ever‐changing health environment (Cowley et al. 2015).

It is to the current challenges facing health visiting that we now turn to capture how June's legacy continues to shape practice and research.

## 5 Current Challenges

As Clark (1976) identified, health visiting practice remains complex and broad, focused on whole families and the neighbourhoods they inhabit. This means that it needs to change continuously because the health needs of populations also evolve constantly. Society and health visiting have changed dramatically since the young June Clark entered the profession some 60 years ago, when evidence for the profession was sparse. If Clark were to enter health visiting now, she would notice the huge amount of evidence about health needs, particularly focused on equity, social justice, and the impact of the early years on later health, as well as on how health visitors can help address those needs.

Health visiting resources and professional direction are influenced by public health policy, as services are commissioned annually by local or national governments. Public health policy, in turn, is influenced by legislation and underpinned by recommendations from public health bodies including the World Health Organization (WHO) and country‐specific Departments of Health. Pertinent public health, health promotion, and safeguarding policy derive from the macro, meso, and micro level perspectives, and they underpin contemporary health visiting practice as well as evidence‐based research.

The most recent Marmot review on health inequalities (Marmot et al. 2020) updated earlier recommendations from 10 years earlier, when six areas of policy change were proposed (Marmot et al. 2010). These included reference to giving children a better start in life, enabling them to reach their potential, creating employment opportunities and promoting healthy living to help in the creation of healthier communities. The recommendations can only be achieved however with investment in public health and health promotion activity by Governments that include investment in the provision of Health visitors.

Health visitors are now particularly well placed to address significant health needs and health inequalities, in the form of early and effective intervention and prevention strategies for babies, children and families. This is because health visiting is the only service in the UK to systematically and proactively reach all babies and children (Institute of Health Visiting (iHV) 2025). There are still nuances about the health visiting role that would be familiar to a young June Clark. Indeed, the idea of health visitors building a relationship with the whole family and local neighbourhood is not only a valued approach but is backed by research that shows it is central to a distinct form of practice orientated towards salutogenesis (health creation), human valuing (e.g., through person‐centred practice) and human ecology (people in their social context) (Cowley and Frost 2006).

However, there has also been a shift to different ways of managing complex caseloads (Whittaker et al. 2021), often involving a wider health visiting team in home visiting, child development reviews, supporting parents with the child's behaviour, speech and language development, toileting, school readiness, and others. Questions arise about whether the delegation of such essential elements of practice enhance or impede the effectiveness of health visiting, but there is a paucity of research to provide us with answers. With developments and team expansion the health visitor role has also changed and now varies across the UK nations, with health visitors facilitating and delegating many aspects of child health programme and taking the lead on the safeguarding of babies, children and their parents/carers. Despite more evidence about the essential role that health visitors play from an early intervention and prevention perspective, the profession is now diminished in numbers, particularly in England. Recruitment into the profession has also been affected negatively and retention of the workforce is challenge given the focus on safeguarding.

The Institute of Health Visiting (iHV) 11th annual survey (iHV 2025) reflected on the health needs of families as well as on workforce pressures within day‐to‐day practice. This survey captured the views of 1392 health visitors from across the UK. Health visitors reported encountering an increased level of need, with obvious markers that are likely to impact children's health and development negatively, such as:

- More parents impacted and struggling with the impact of poverty,
- The increased use of food banks,
- An increase in the families skipping meals because of the cost‐of‐living crisis,
- An increase in perinatal mental illness,
- A rise in domestic abuse,
- An increase in homelessness,
- More individuals seeking asylum.

The stress associated with poverty impacts early brain development which subsequently affects child development. Marmot et al. (2020) clearly makes this connection. Not addressing health inequality will have a negative effect on babies and children reaching their full potential. Health visitors are observing this impact daily, with inequalities widening and child health deteriorating, and with that more children being exposed to adverse risks.

The iHV (2025) survey also reports that health visitors are seeing more children with speech, language and communication delays, more children with behavioural and child development problems and a reported increase with children with additional needs (such as autism/signs of autism). Some children are not ready for school with delays in toileting, knowing how to play, responding to other children and being confident in sharing (Kindred^2^ 2025). Fixing health inequality lies with Government, but the day to day support of babies, children and families fall within the role of the health visitor. For the role to be delivered in its fullest form, where every contact is delivered as intended within the UK healthy child programmes, there needs to be more investment into the role and recognition of it potential. These same arguments were posed by June Clarke throughout her career.

Health visitor workforce numbers are continuing to fall (iHV 2025). More health visitors from England within the iHV survey felt able to provide families with ‘continuity of health visitor’ ‘all or most of the time’ compared to the other UK nations. Health visitors reported struggling to address health needs due to lack of investment in their service. This will only have a detrimental impact in the future if this shortfall in health visiting is not addressed.

Nurturing care is essential for early childhood development (WHO 2018) and health visitors aim to build trusting, nurturing, and therapeutic relationships when they are given time, space and investment to do so. This builds parents' confidence, allows risk assessment to be carried out when needs are unmet and signposting to the appropriate intervention.

Some health visitors report burnout and stress; finding it difficult to ‘switch off’ due to the emotional burden that the role brings, with only 46% of English health visitors reporting confidence in meeting the needs of babies and children who were deemed vulnerable, ‘all or most of the time’ (iHV 2025, 15). This is a concerning finding. Safeguarding risk assessment is now an essential part of the role and identifies the need for supervision policies and delivery models to support health visitors in their safeguarding practice, as well as provision of restorative clinical supervision to support challenges facing the workforce. These issues are multi‐faceted and mean that the health visiting workforce is under pressure and work‐related stress is on the increase. Despite these challenges, Alison Morton, Chief Executive Officer at the iHV, says ‘It is not too late to turn this situation around’ (iHV 2024). With investment, the health visiting service has the potential to impact our future generations positively, it is fixable and recognised as ‘the backbone of early years… and considered a ‘safety net’ around all families’. (UNICEF UK 2022, 21).

## 6 Concluding Reflections

Professor Dame June Clark's intellectual leadership and her willingness to challenge accepted orthodoxies left an indelible mark on health visiting and on nursing more broadly. June showed how rigorous scholarship could articulate the distinctive contribution of health visiting, and in doing so, she created the foundations for a discipline that continues to seek recognition and renewal. Her early paper in the very first issue of the *Journal of Advanced Nursing* is an important example of how new ideas can ignite enduring change. That work was far more than a study of practice in Berkshire: it was a spark that helped launch subsequent innovations in health visiting education, research, and service development. By situating health visiting within a broader intellectual and professional context, June created space for others to imagine, test, and evaluate new approaches, and this is work that continues to shape the profession today.

Reflecting on June's professional life reminds us of the critical role that leaders can play in shaping professional identity, influencing policy, and sustaining debate across national and international arenas. Her work consistently sought to show how nursing and health visiting mattered, and were vital contributions to public health, equity, and social justice. June combined formidable courage with a profound sense of professional responsibility, and in doing so, she inspired others to follow her lead.

Her legacy is not only in the multiple titles she held, or the many honours bestowed, but in her enduring conviction that nursing and health visiting could transform the lives of children and families; and, in the vision she offered for a fairer, healthier society in which children and families are supported from the earliest days of life. June was a disruptor, a visionary, and a nurse scholar who nurtured others to find their scholarly voice. At a time when nursing and health visiting face workforce shortages, widening inequalities, and shifting policy landscapes, her example is both timely and inspiring. The ‘exquisite obsession’ that drove June Clark's life's work remains a call to action: to continue advocating, researching, and innovating so that nursing and health visiting fulfill their promise as cornerstones of public health and social justice.

## Conflicts of Interest

The authors declare no conflicts of interest.

## Data Availability

Any data cited in this article are available from the authors.
